# Life‐threatening laryngeal injury in Elite Rugby Union: Prevention and management laryngeal trauma in rugby

**DOI:** 10.1002/ccr3.3565

**Published:** 2020-11-20

**Authors:** Bamidele Famokunwa, Simon Kemp, Julia Selby, Gitta Madani, Guri Sandhu, James H. Hull

**Affiliations:** ^1^ Department of ENT Wexham Park Hospital Slough UK; ^2^ Rugby Football Union Twickenham UK; ^3^ Department of Respiratory Medicine Royal Brompton Hospital London UK; ^4^ Department of Radiology Charing Cross Hospital London UK; ^5^ Department of ENT Charing Cross Hospital London UK

**Keywords:** breathing, fracture, larynx, rugby, sport, trauma

## Abstract

Laryngeal trauma is a life‐threatening injury in contact sports. Due to its potentially devastating consequences, the prevention, diagnosis, and management of neck trauma both pitch side and at the hospital are essential for athletes.

## Graphical abstract

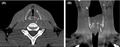

**KEY POINTS**

- Laryngeal injury is a life‐threatening risk in contact sports carries and has severe morbidity.
- Exercise‐induced laryngeal obstruction (EILO) can occur as a sequelae to trauma.
- Athletes can return to monitored training within 2 months of laryngeal surgery.
- Due to the rarity and complexity of laryngeal trauma in sport, effective management requires a multidisciplinary approach for acute in‐field management, hospital care, and the rehabilitation phase of recovery.
- Future work should focus on improving understanding and then managing the risk of traumatic laryngeal injury in contact sports.

## 1 INTRODUCTION

Laryngeal trauma is a life‐threatening injury resulting from above contact to the throat/anterior neck and can cause acute upper airway compromise with fatal consequences. It accounts for between 1 per 14 000 and 30 000 accident and emergency attendances and blunt laryngeal injuries represent 0.22%‐1% of presentations seen in major urban trauma centers.^1^, ^2^, ^3^, ^4^ It is postulated that approximately one in 2500 trauma resuscitations involve compromise of the upper aerodigestive tract.^1^ It is reported that worldwide among sports, football and rugby account for the majority of traumatic neck injuries.^5^ Due to its potentially devastating consequences, the prevention, diagnosis, and management of neck trauma both pitch side and at the hospital are essential for athletes. Through this case, we show that effective management requires a multidisciplinary approach for acute in‐field management, hospital care, and the rehabilitation phase of recovery.

## 2 CASE REPORT

**FIGURE 1:**
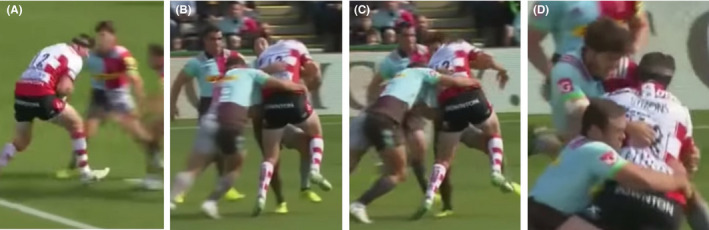
Tackle sequence (A) prior to the contact being made, (B) initial contact of left shoulder into neck, (C) shoulder rides up into face, (D) players fall to ground. Link to video of injury —https://www.youtube.com/watch?v=TodNq3aGFzk

In September 2017, a 28‐year‐old male international rugby player sustained direct trauma to the left side of his neck during an English Premiership match (Figure 1 and see https://www.youtube.com/watch?v=TodNq3aGFzk).

He was immediately apneic for a few seconds and aphonic within 1 minute of the tackle, rapidly developing respiratory compromise. He was given basic airway support with supplementary oxygen and transferred to hospital. He reported immediate panic when he realized that while he was in immediate respiratory distress, he was also unable to adequately communicate.

**FIGURE 2:**
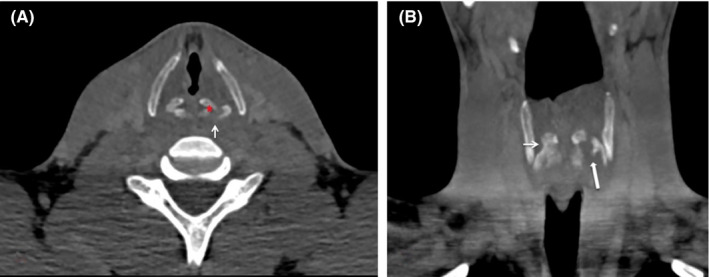
(A) Axial CT demonstrating a fracture of the left posterior cricoid cartilage (arrow). Note the arytenoid cartilage (*) is interposed between the displaced cartilage fragments, (B) coronal maximum intensity projection (MIP) reconstruction demonstrating the widely separated fracture of the left posterior cricoid cartilage (block arrow) resulting in disruption of the cricoarytenoid joint. Note the normal right cricoarytenoid joint (arrow)

**FIGURE 3:**
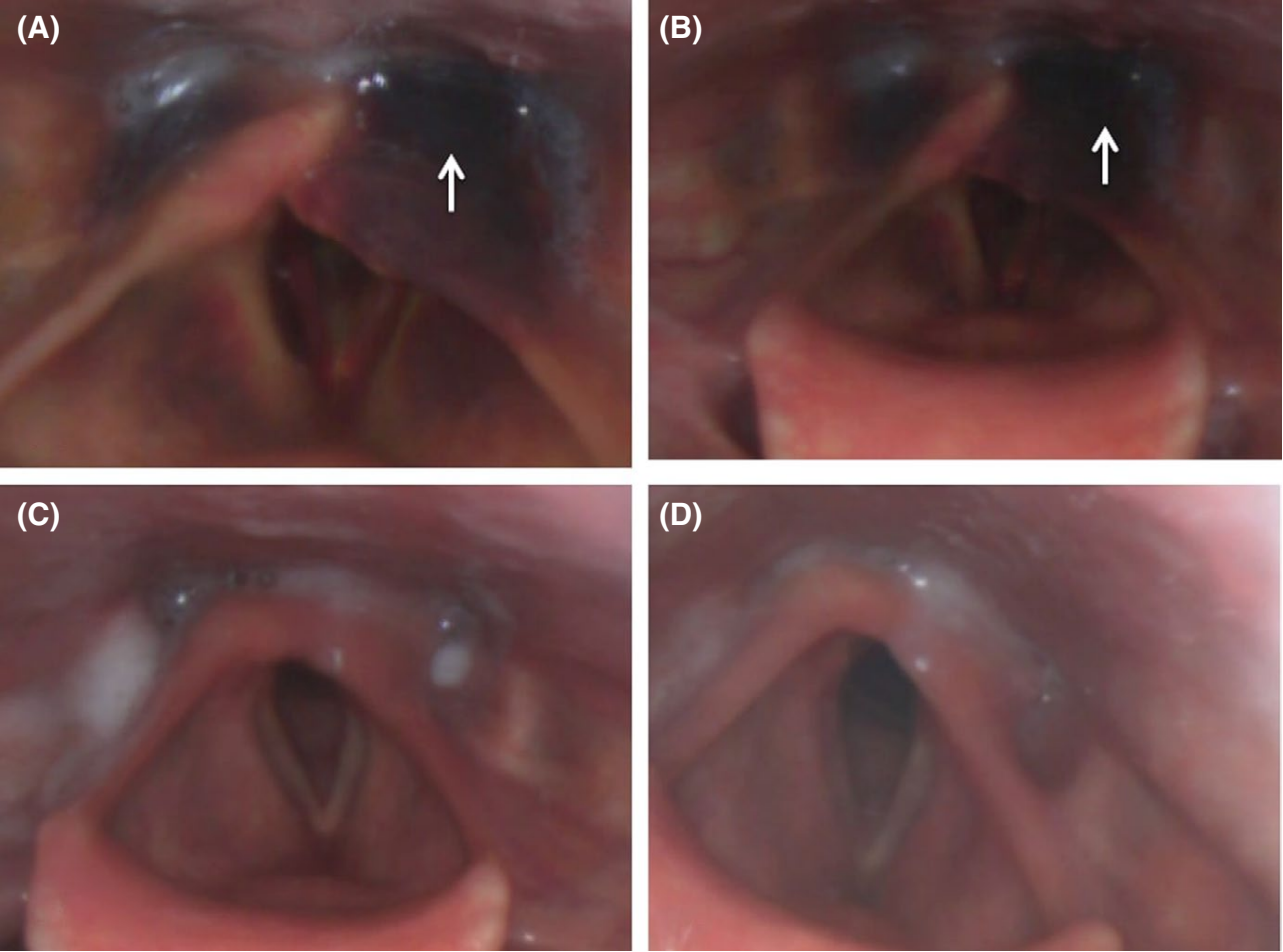
Laryngoscopic appearances on (A, B) day 6 revealing acute hemorrhage (arrow) and edema similar to findings at presentation, (C) 6 wk postarytenoidectomy, (D) 4 mo postarytenoidectomy

Once stabilized, computer tomography (CT) imaging revealed a fracture of the posterior cricoid cartilage into the left arytenoid (Figure 2). Direct laryngoscopic visualization revealed edema and bruising (Figure 3A). Using the Becker (modified Schaefer) classification of laryngeal trauma, the injury was graded as a group 2.^6^ Becker group 2 laryngeal injuries have more severe edema, hematoma, and minor mucosal disruption without exposed cartilage. The fracture was managed conservatively with intravenous steroids and clinical surveillance in a high dependency unit, mandating a week‐long stay in hospital.

### 2.1 Subsequent clinic management

Specialist upper airway speech and language therapy (SLT) assessment 1 week after injury revealed turbulence on inspiration, a weak, breathy voice, and swallowing impairment. Initial therapy focused on voice conservation and postural adjustments to avoid further strain and facilitate healing, use of diaphragmatic breathing patterns combined with exercises to encourage vocal fold abduction.

Over eight therapy sessions (one session every 3‐4 days), the intensity of laryngeal abduction and forward resonance exercises was increased with improvements to both voice and respiratory function. Biofeedback laryngoscopy was used to improve laryngeal control of breathing.

### 2.2 Management of trauma‐induced exercise‐induced laryngeal obstruction

Despite intensive therapy, the patient remained breathless on exertion. A diagnosis of exercise‐induced laryngeal obstruction (EILO) secondary to trauma was suspected. Continuous laryngoscopy during exercise (CLE) test^7^ was performed and revealed a prolapse of the left arytenoid, which increased with exercise intensity.

Surgery is a treatment option for EILO that does not respond to conservative measures.^8^ He subsequently underwent a left arytenoidectomy 5 weeks after the injury. Postoperative therapy was tailored to support a phased return to fitness training. He made his return to training 1 month after surgery.

### 2.3 Return to play

The SLT attended training sessions as exercise intensity increased. Safe projection work encompassed breath support and subtle modifications to resonance, pitch, and articulation to decrease laryngeal loading. At 8 weeks following laryngeal surgery, there was no compromise to breathing on exertion associated with a heart rate 160 bpm and there was no discernible deficit in vocal function. He returned to professional play in March 2018, 6 months after the initial injury.

The CARE case report guidelines were used when writing this report.

## 3 DISCUSSION

Blunt laryngeal trauma can have immediately life‐threatening consequences^9^ which highlights the importance of sports clinicians having a heightened awareness of this problem.

### 3.1 Multidisciplinary approach to management

#### 3.1.1 On‐field management

Insuring that on‐field medical staff have the appropriate competencies in immediate care in sport is a key element of secondary injury prevention programs. In English Premiership matches, medical staff must have completed a minimum of a yearly level three course in Immediate Care in Rugby. This includes advanced airway management with training in needle cricothyroidotomy and the surgical airway.

#### 3.1.2 In hospital—conservative vs surgical

The indication, timing, and nature of surgical intervention have been largely dictated based on single‐center case series. There is thus great heterogeneity with the surgical approach.^9^, ^10^ Bent et al^11^ reviewed 77 patients with laryngeal trauma and concluded that conservative treatment of Schaefer group 1 and 2 injuries was 100% effective and surgical repair within 48 hours for those requiring surgery improved outcomes.

Our patient was classified as Becker group 2, with no indication for early surgical intervention. The later development of trauma‐induced EILO necessitated arytenoidectomy. EILO is a cause of exertional breathing difficulties.^12^ The vast majority of cases are caused by supraglottic closure of the laryngeal inlet.^13^ It is diagnosed using CLE,^7^ which allows visualization of the larynx during incremental bouts of exercise. Treatment options vary greatly by center and include specialist SLT for laryngeal control, exercise therapy with a physiotherapist for breathing pattern work and surgery.^14^ Due to the severity of the EILO and the lack of full symptom resolution with conservative treatment, our patient was deemed to be a candidate for surgery. We believe this to be the first case of trauma‐induced EILO treated with arytenoidectomy.

#### 3.1.3 Longer‐term follow‐up

Successful longer‐term management of this type of injury is dependent on high‐quality multidisciplinary team (MDT) input. Following arytenoidectomy, flow parameters improved but were still attenuated to 66% of predicted values. SLT therefore provided tailored breathing techniques to maximize laryngeal aperture during exercise. Deficits in voice and respiratory function may only develop when exercise intensity increases so it is important that specialist therapy is integrated with physical training sessions.

Our patient began training within 6 weeks of surgery and this speed of return to sport is similar to the figures seen in a case series of patients that had surgery for EILO.^14^

### 3.2 Considerations for safety in rugby

Recent changes to tackle laws in rugby should act to reduce risk of blunt laryngeal trauma. Specifically, in addition to enforcing current rules, World Rugby trialed a new tackle height (the level of the armpit), during the start of the 2018 Championship Cup season and tacklers made contact with the ball carrier's head and neck 30% less often.^15^

## 4 CONCLUSION

In conclusion, blunt laryngeal trauma is a risk in contact sports such as rugby and more extensive research is needed to evaluate its incidence within the sport. The care of athletes who have sustained such an injury is complex and it is mandatory to have a plan in place to manage the acute in‐field event and the multidisciplinary team rehabilitation phase. Future work should focus on improving understanding and then managing the risk of traumatic laryngeal injury in contact sports.

## CONFLICT OF INTEREST

None declared.

## AUTHOR CONTRIBUTIONS

BF: prepared this case report for scientific publication. SK: provided details on the on‐field management of rugby injuries. JS: provided specialist speech and language therapy and contributed to the drafting of the manuscript. GM: involved in the acquisition and reporting of the imaging. GS: led the surgical care of the patient. JH: led in the clinical care of the patient and contributed substantially to the contents of the final version of this manuscript.

## ETHICAL APPROVAL

The informed consent was obtained from the patient for the presentation of this case.

## Data Availability

The data that support the findings of this study are available on request from the corresponding author. The data are not publicly available due to privacy or ethical restrictions.
